# *Origanum majorana* L. Essential Oil-Associated Polymeric Nano Dendrimer for Antifungal Activity against *Phytophthora infestans*

**DOI:** 10.3390/ma12091446

**Published:** 2019-05-04

**Authors:** Vu Minh Thanh, Le Minh Bui, Long Giang Bach, Ngoc Tung Nguyen, Hoa Le Thi, Thai Thanh Hoang Thi

**Affiliations:** 1 Institute of Chemistry and Materials, 17 Hoang Sam, Cau Giay, Hanoi 100000, Vietnam; vmthanh222@yahoo.com; 2 NTT Hi-Tech Institute, Nguyen Tat Thanh University, 300A Nguyen Tat Thanh, District 4, Ho Chi Minh City 700000, Vietnam; blminh@ntt.edu.vn (L.M.B.); blgiang@ntt.edu.vn (L.G.B.); 3 Center of Excellence for Functional Polymers and NanoEngineering, Nguyen Tat Thanh University, Ho Chi Minh City 700000, Vietnam; 4 Center for Research and Technology Transfer (CRETECH), Vietnam Academy of Science and Technology, 18 Hoang Quoc Viet, Cau Giay District, Hanoi 100000, Vietnam; tungnguyen.vast@gmail.com; 5 Biomaterials and Nanotechnology Research Group, Faculty of Applied Sciences, Ton Duc Thang University, Ho Chi Minh City 758307, Vietnam; mytom.61303531@gmail.com

**Keywords:** antifungal activity, dendrimer, *Origanum majorana* L. essential oil, *Phytophthora infestans*

## Abstract

In this study, the introduction of *Origanum majorana L.* essential oil into a polyamidoamine (PAMAM) G4.0 dendrimer was performed for creation of a potential nanocide against *Phytophthora infestans*. The characteristics of marjoram oil and PAMAM G4.0 was analyzed using transmission electron spectroscopy (TEM), nuclear magnetic resonance spectroscopy (^1^H-NMR) and gas chromatography mass spectrometry (GC-MS). The success of combining marjoram oil with PAMAM G4.0 was evaluated by FT-IR, TGA analysis, and the antifungal activity of this system was also investigated. The results showed that the antifungal activity of oil/PAMAM G4.0 was high and significantly higher than only PAMAM G4.0 or marjoram essential oil. These results indicated that the nanocide oil/PAMAM G4.0 helped strengthen and prolong the antifungal properties of the oil.

## 1. Introduction

In Vietnam, tomato is ranked tenth in terms of crop value, topping at more than 9.7 billion VND in 2005. However, tomato late blight, caused by *Phytophthora infestans*, is a destructive disease that causes heavy decline in tomato production for many years. According to the General Statistics Office of Vietnam, the late blight caused by *Phytophthora infestans* has drastically hindered the growth of tomato cultivation area in Vietnam from 2009 to 2012, resulting in a halt in production yield [1]. Studies on the distribution as well as the effects of the late blight disease have also been conducted very early. According to the assessment of harm caused by late blight in the suburbs of Hanoi in 1965, the average loss of 30–70%, at the high level, can cause complete loss of productivity. In recent years, the level of the disease is still high. Severe outbreaks of late blight have been observed in many suburbs in Vietnam [1,2]. 

Recently, fungicides of biological origin have been strongly accentuated as it could overcome the inherent limitations of chemical pesticides. In addition, the approach of using antifungal substances extracted from plants was reported to be a promising agent against fungi. Marjoram (*Origanum majorana* L., Lamiaceae) is a perennial species originating in southern Europe. The plant has been widely cultivated and used in cooking as a spice. More importantly, distillates from marjoram are highly valued for its antimicrobial, antifungal and antioxidant activity. In one study, marjoram oil has been tested against various bacterial and fungal species, demonstrating improved effectiveness against *Beneckea natriegens*, *Erwinia carotovora* and *Moraxella* [3]. In addition, marjoram oil has been studied extensively in the field of plant disease control, especially against *P. infestans.* It is considered to be a fungicidal alternative to chemically derived drugs [4]. Besides marjoram essential oil, *P. infestants* was reported to be inhibited by pathogen-induced proteins in previous studies [5,6]. However, the process for utilizing the pathogen is quite complicated and not suitable for excess products. When it comes to combating microbes, the use of essential oils has various advantages. First, since essential oils are of natural origin, it is completely safe for humans and the environment. Second, since essential oils are mixtures of many different compounds, the use of essential oil as an antimicrobial agent could impair the adapted resistance against drugs of microbes in various ways. 

However, essential oil from herbal sources demonstrates many disadvantages including poor stability, solubility, and volatility. One of the approaches to remedy such limitations is the application of nano-encapsulation, which is capable of reducing volatility, improving the stability, water solubility, and efficiency of essential oil-based formulations, while still maintaining therapeutic efficiency of the drug [7,8,9,10,11]. Dendrimer is a spherical, branched, nanoscale material that is more prominent than linear polymers. The dendrimer consists of three parts including core, inner branches, and lateral groups. Among dendrimers, polyamidoamine (PAMAM), a group of branched-chain dendrimers, including amine branching and amide bridging, is the most widely used dendrimer due to its amine functional groups (for even-numbered PAMAMs) and carboxylates (for odd-numbered PAMAMs) that help dissolve dendrimer in polar solvent [12,13]. These groups are also very active so it is easy to react to create new structurally diverse substances [14]. One of the important uses of PAMAM G4.0 (polyamidoamine generation 4) is acting as a carrier system for the transport of biological molecules and drugs for the treatment of cancer diseases [15,16]. Recently, many studies in using dendrimer in biocide has been proved to be much potential [17,18,19,20]. For example, Winnicka and co-workers reported the capability of PAMAM dendrimer in increasing the antifungal activity of clotrimazole against different strains of *Candida* [19]. Later, Winnicka continued to design a mixture containing PAMAM G2.0 and ketoconazole that also dramatically enhances the antifungal action of the drug [20]. In another study, Jose and co-workers employed different generation of PAMAM dendrimer (G1.0–G3.0) as agents to improve water solubility of amphotericin B drug, thereafter enhancing its antifungal action [21]. However, there are not much reports in combining extracted essential oil with dendrimer as carrier system. 

This study attempted the combination of marjoram essential oil with PAMAM G4.0 to produce the oil/PAMAM G4.0 dendrimer system. The system was then evaluated for preservation efficiency of natural oil functions and tested for antifungal activity against *P. infestans*. The polymer structure and morphology of the PAMAM G4.0 were characterized. TGA analysis and the FT-IR spectrum of PAMAM G4.0 containing this volatile oil were also examined.

## 2. Materials and Methods 

### 2.1. Materials

Ethylenediamine (EDA) and toluene were purchased from Merck (Darmstadt, Germany). Methyl acrylate (MA) was purchased from Sigma-Aldrich (St. Louis, MO, USA). Methanol was supplied by Fisher Scientific (Houston, TX, USA). Spectra/Por^®^ Dialysis Membrane (MWCO 3.5 kDa) was purchased from Spectrum Laboratories Inc. (Rancho Dominguez, CA, USA). Marjoram essential oil was supplied by NTT Hi-Tech Institute, Nguyen Tat Thanh University, Ho Chi Minh City, Vietnam. *Phytophthora infestants* fungi strain was purchased from Gia Tuong Ltd, Vietnam. Ethanol and acetic acid were purchased from Xilong Chemical, Ltd. (Guangdong, China). All other chemicals were of reagent grade. Distilled water was used in all preparations.

### 2.2. Methods

#### 2.2.1. Preparation of PAMAM G4.0 Dendrimer

PAMAM dendrimer generation 4.0 (PAMAM G4.0) was synthesized from the EDA core utilizing divergent approach as previously reported in our literature [13], in which the EDA’s primary amine groups react with MA’s acrylate groups via Michael addition reaction to form half generation PAMAM, followed by the reaction between half generation PAMAM’s methyl propionate groups with excess EDA to form full generation PAMAM dendrimer, denoted by Gn.0. Briefly, EDA (20 mL) was added to 150 mL of MA dissolved in methanol. The reaction was in turn kept under stirring for 3 h at 0 °C and then 48 h at room temperature. The removal of impurities and solvent was performed using rotary vacuum evaporator (Strike 300, Lancashire, UK), resulting in the core precursor G0.5. Next, G0.5 was added to EDA solution (130 mL) to obtain PAMA G0.0. The mixture was stirred for 96 h at room temperature, rotated under vacuum using mixed solvent (toluene: methanol is 9:1 v/v). Finally, the resulting mixture was dialyzed by dialysis membrane against methanol to remove excess toluene and EDA and dried under vacuum to remove methanol. This protocol was repeated continuously to obtain PAMAM G4.0.

#### 2.2.2. Characteristics of Dendrimer PAMAM G4.0

The chemical structure of dendrimer PAMAM G4.0 was analyzed using Nuclear Magnetic Resonance spectroscopy (Bruker Advance 500, Bruker Co., Billerica, MA, USA). Deuterated chloroform was used as solvent. For illustration of size and morphology, Transmission Electron Spectroscopy (JEM-1400 TEM; JEOL, Tokyo, Japan) was utilized. A drop of sample solution prepared in distilled water was placed on a carbon–copper grid (300-mesh, Ted Pella Inc., Redding, CA, USA) and air dried in 10 min for the TEM observation. 

#### 2.2.3. Evaluation of the Composition of Marjoram Essential Oil

The composition of the marjoram oil was determined by Gas Chromatography Mass Spectrometry (Agilent Technologies, Santa Clara, CA, USA). 

#### 2.2.4. Synthesis of PAMAM G4.0 Combining Marjoram Essential Oil

A rotating system was used to drain out all water vapor in the PAMAM G4.0 dendrimer to ensure that PAMAM G4.0 is completely dry. Marjoram essential oil (500 μg, 1000 μg, 2000 μg, and 5000 μg) was quickly added to PAMAM G4.0 according to the ratios (1:1). After being ultrasonicated for 15 min at room temperature, the samples were rotated at 100 °C for 2 min, allowing the oil molecules combine with the dendrimer structure. Samples were stored in refrigerator for later use.

#### 2.2.5. Characterization of Oil/PAMAM G4.0

Marjoram oil, PAMAM G4.0 and the combined system were analyzed with Spectrum Tensor27 FT-IR Spectrometer, using KBr pellets FT-IR Spectrometer in the range of 400–4000 cm^−1^. All samples were mixed with KBr and pressed into a pellet before the measurement. Thermal gravimetric analysis (TGA) of marjoram essential oil, PAMAM, and oil/PAMAM were carried out using TG Analyzer (Mettler Toledo, Culumbus, OH, USA).

#### 2.2.6. Agar diffusion method

The antifungal effect of marjoram oil, PAMAM G4.0 and the system were assessed using agar diffusion method. The PDA (Potato Dextrose Agar) agar plates were prepared by potato infusion at 250.00 g/L, glucose at 20.00 g/L, and agar at 20.00 g/L. After that, fungal solution was prepared and obtained the density of 6.0465 × 10^8^ CFU/mL after 48 h of incubation. The solution was then diluted 2 times, followed by the inoculation of 100 µL diluted fungal solution on the agar surface. Appropriate amount of synthesized oil/PAMAM sample was dissolved in 1 mL of distilled water first to get the desired final concentration, which are 500 ppm, 1000 ppm, 2000 ppm, and 5000 ppm. Next, 100 µL of sample solution was added to each well of 6 mm in diameter. After 48 h, the inhibition zone was measured in diameter. The data were expressed as mean ± SD.

## 3. Results and Discussion

The preparation of PAMAM dendrimer generation 4.0 (G4.0) was obtained from the initiator EDA core using two-step synthesis in which the odd steps yield half generation PAMAM followed by the even steps which generate full generation PAMAM dendrimer.

### 3.1. Chemical Structure of Synthesized Dendrimer PAMAM G4.0 

**Figure 1:**
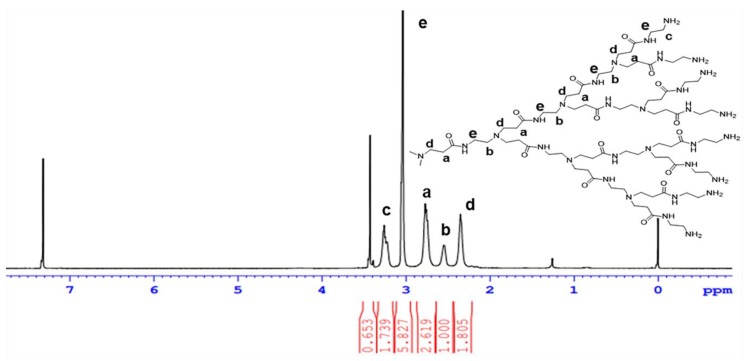
^1^H NMR spectrum of PAMAM G4.0.

The spectra of ^1^H NMR proton of PAMAM G4.0 is shown in Figure 1. As shown in Figure 1, the protons at 2.351 ppm, 2.55 ppm, 2.77 ppm, 3.04 ppm and 3.23 ppm were assigned to protons in methylene groups of –CH_2_–CO–NH (peak d), CH_2_–N– (peak b), –CH_2_–NH_2_– (peak a), –N–CH_2_–CH_2_– (peak e), and –CO–NH–CH_2_ (peak c). The presence of all these signals demonstrated that PAMAM G4.0 was successfully synthesized and characterized based on other studies in NMR characterization of fourth-generation PAMAM [13,16].

### 3.2. Characteristics of PAMAM G4.0 and Oil/PAMAM G4.0

**Figure 2:**
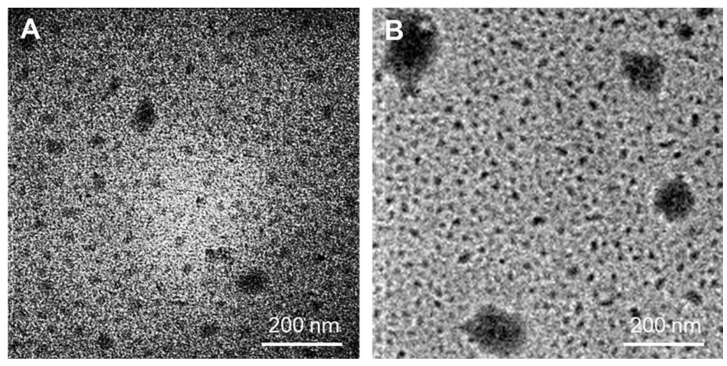
TEM image of polyamidoamine dendrimer PAMAM G4.0 (**A**) and oil/PAMAM G4.0 (**B**).

Figure 2 displays the morphology and particle size of PAMAM G4.0 dendrimer. Since the eradication of the fungi depends on the transportation of marjoram essential oil, it is more likely for the dendrimer to penetrate into the spore or mycelium (the size of the spore ranges from 19 × 10^−12^ to 23 × 10^−12^ mm) as its size gets tinier [3]. Analysis of TEM results showed that dendrimer PAMAM G4.0 was 20 to 30 nm in size and fairly uniform. There are some aggregates (<70 nm) were shown to occur but in low frequency. After the oil was associated with dendrimer, a significant increment in the diameter of obtained oil/PAMAM G4.0 (40–150 nm) was observed, indicating the successful combination of essential oil with PAMAM G4.0 structure. There are many studies reported that the particle size of less than 500 nm is sufficiently good for the penetration of nanocarriers into fungal cells [22,23,24,25]. As a result, G4.0 and oil/PAMAM G4.0 is suitable for associating with oil to fully interact with the fungus’s cell membrane as well as easily penetrates into the cell layer, thus destroying fungal cells. 

### 3.3. Evaluation of the Composition of Marjoram Essential Oil

**Figure 3:**
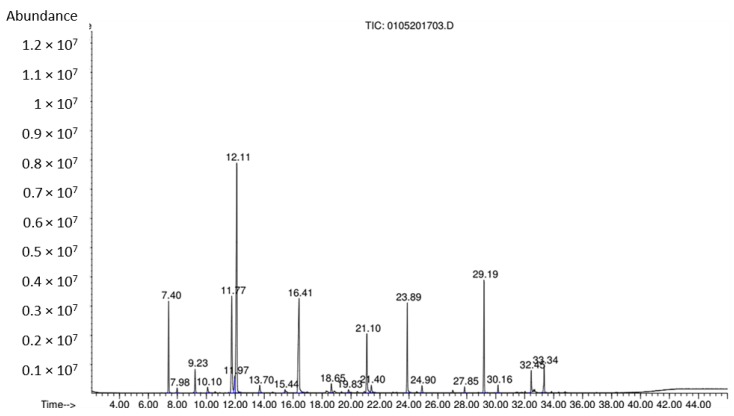
The GC-MS spectrum of marjoram essential oil.

**Figure 4:**
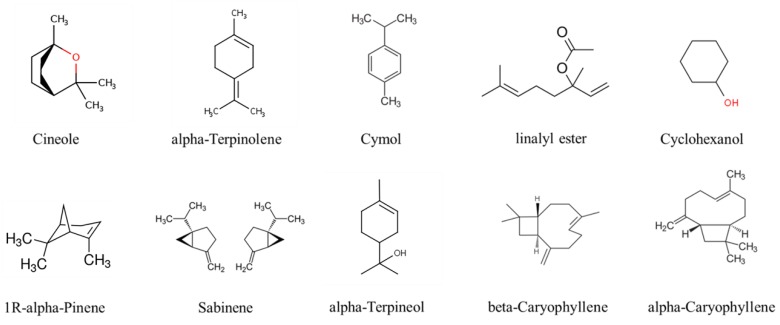
Chemical structures of marjoram oil’s components.

Figure 3 shows the composition and content of the substances in marjoram essential oil analyzed by GC-MS. From the GC-MS analysis, it is revealed that major components in the oil are 1R-alpha-pinene, sabinene, cymol, cyclohexanol, cineole, alpha-terpinolene, alpha-terpineol, linalyl ester, beta-caryophyllene, alpha-caryophyllene. Chemical structures of these components of marjoram oil are shown in Figure 4. Cineole (28.59%) is the predominant constituent in the oil, followed by alpha-terpinolen (14.75%) and cymol (11.82%). Compared to the analysis results of other studies, the reported composition of the oil is similar. However, component contents are different, possibly due to the difference in cultivation process, the habitat of the plant, the extraction and preservation method [4]. 

### 3.4. FT-IR Analysis

**Figure 5:**
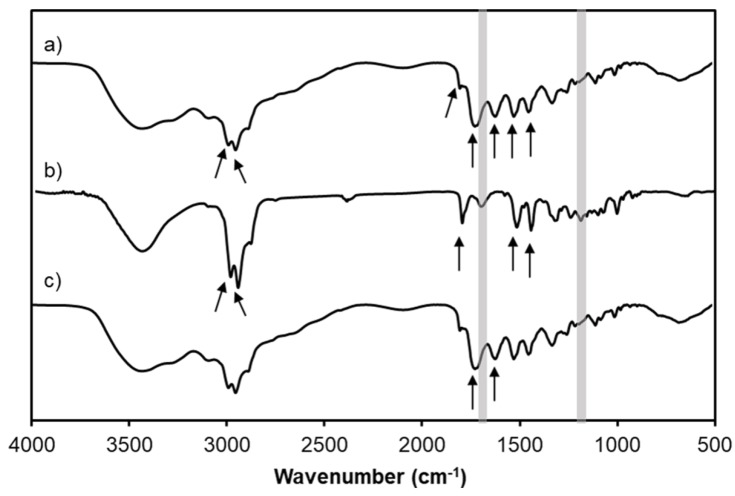
FT-IR spectra of (**a**) oil/PAMAM G4.0; (**b**) Marjoram essential oil and (**c**) PAMAM G4.0.

Figure 5 demonstrates FT-IR spectra of marjoram essential oil, PAMAM G4.0 and oil/PAMAM G4.0. The characteristic peaks of PAMAM G4.0 at positions 1661 cm^−1^ and 1553 cm^−1^ are the signals of the (-CO-NH-) bonds of grades I and II, respectively, which were also observed clearly on the spectrum of oil/PAMAM G4.0. Five characteristic peaks of the essential oil at position 2967 cm^−1^; 2927 cm^−1^; 1740 cm^−1^; 1451 cm^−1^; and 1376 cm^−1^ have also been observed clearly on the spectrum of oil/PAMAM G4.0. Signaling of the essential oil and dendrimer PAMAM G4.0 were both observed in the spectrum of the synthesized oil/PAMAM G4.0 sample, indicating that the synthesis of dendrimer PAMAM G4.0 associating marjoram oil was successful. Additionally, there is a disappearance of carbonyl peak at 1700 cm^−1^ in oil/PAMAM G4.0 spectrum as compared to essential oil spectrum. C-O absorption band at 1300 cm^−1^ of alcohol functional groups of essential oil’s components is not detected as well, indicating the formation of hydrogen bonds between essential oil’s components and PAMAM G4.0.

### 3.5. TGA Analysis

**Figure 6:**
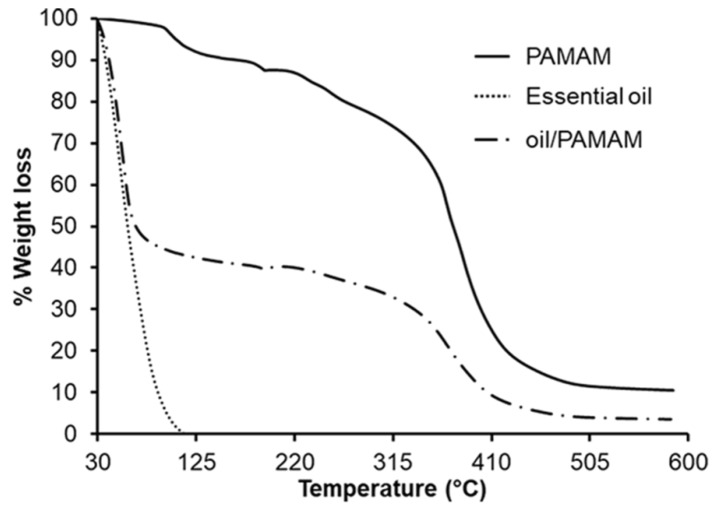
TGA curves of PAMAM G4.0, marjoram essential oil, and oil/PAMAM G4.0.

The actual amount of marjoram essential oil mixed with PAMAM G4.0 was estimated by TGA analysis (Figure 6). The experimental temperature was ramped to 200 °C with heating rate at 3 °C/min and from 200 °C to 600 °C with heating rate at 50 °C /min. The weight loss from room temperature to 100 °C corresponds to the evaporation of marjoram oil. As shown in TGA curves of oil/PAMAM G4.0 with 1000 μg essential oil, the weight loss at 100 °C was around 56%. This means the actual amount of essential oil successfully mixed with 1000 μg PAMAM G4.0 in the final product was about 560 μg. This result indicated the existence of essential oil in oil/PAMAM G4.0 sample. 

### 3.6. Antifungal Experiment

**Figure 7:**
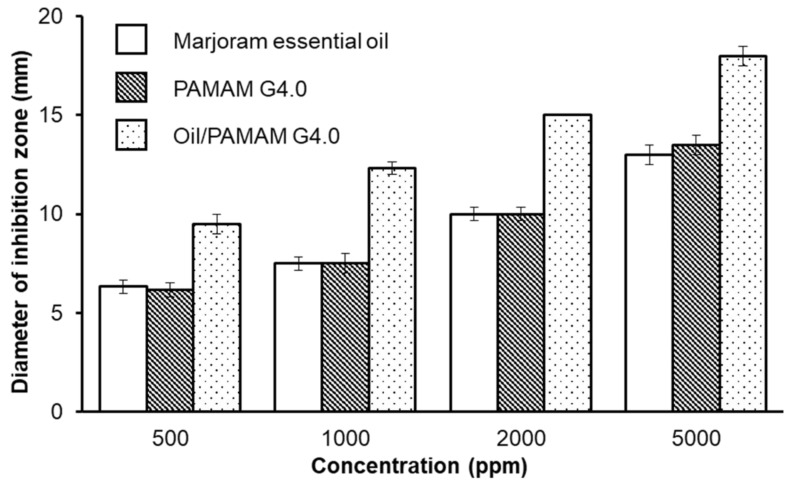
The graph concluding average inhibition zone (d. mm) against *Phytophthora infestants* of marjoram essential oil, PAMAM G4.0 and oil/PAMAM G4.0 based on different concentrations: 500, 1000, 2000 and 5000 ppm.

**Figure 8:**
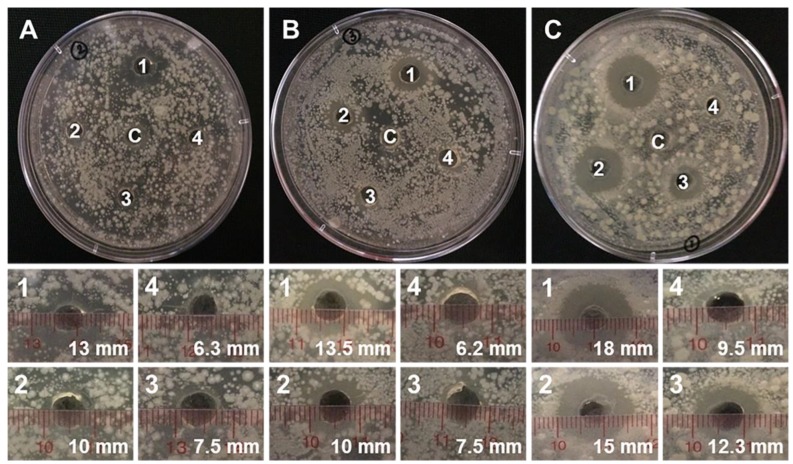
Diameters of inhibition zone of (**A**) Marjoram essential oil; (**B**) dendrimer PAMAM G4.0; (**C**) oil/PAMAM G4.0 after 48 h with different concentrations: (1) 5000 ppm; (2) 2000 ppm; (3) 1000 ppm: (4) 500 ppm.

Anti-*Phytophthora infestants* properties of the essential oil, PAMAM G4.0 and the mixture of these two compounds at concentrations of 500, 1000, 2000 and 5000 ppm are displayed and summarized in Figure 7, Figure 8 and Table 1. The combination of PAMAM G4.0 and volatile marjoram exhibits superior antifungal properties compared to the marjoram essential oil and PAMAM G4.0. At the lowest test concentration (500 ppm), PAMAM G4.0 has a diameter of inhibition zone of 6.16 ± 0.34 mm. PAMAM G4.0 anti-fungal properties are due to the fact that their surface containing –NH_2_– positively charged particles which could interact and destroy negatively charged microbial membranes. It is also shown that the antifungal property of the essential oil is minimal, as demonstrated by the very small diameter of inhibition zone of 6.33 ± 0.33 mm at 500 ppm concentration and the presence of mycelium around the well. In other words, there are no significant inhibition at both 500 and 1000 ppm of essential oil and PAMAM G4.0, individually. This is contrasted by wells of oil/PAMAM G4.0 at all concentrations, showing recognizable inhibition zones. At higher concentrations of 1000, 2000 and 5000 ppm, the antifungal activity of PAMAM G4.0 and oil, individually, was gradually increased and the strongest activity against *P. infestans* was achieved by the oil/PAMAM G4.0. In other words, oil/PAMAM G4.0 was found to have stronger antifungal ability than that of each individual component. After a series of experiments, it can be concluded that the concentration of oil/PAMAM G4.0 at 500 ppm, which respectively exhibited 10 and 22 times bigger inhibition diameter (9.50 ± 0.50 mm) than that of essential oil and PAMAM G4.0 at the same concentration (500 ppm), provided the optimal antifungal result as compared to other combinations of PAMAM G4.0 and oil. At higher concentration (5000 ppm), the synergistic effect seemed to be lost. This could be explained by the reason that not all marjoram essential oil used was successfully mixed with PAMAM G4.0, as revealed in TGA analysis. Due to steric hindrance effect, reduced ability of essential oil to be mixed and combined with PAMAM as the concentration of PAMAM increased. In addition, the diffusion of mixed essential oil from the mixture oil/PAMAM G4.0 out of the well to the agar surface in agar diffusion test could also be restricted with the increasing of PAMAM molecules concentration. Taken together, thanks to the synergy between PAMAM G4.0 and oil, not only the required concentration for each component was reduced significantly, it was also possible to maintain the sufficient result, which is important for reduction of environmental pollution risk.

As revealed in GC-MS result, marjoram essential oil comprised several components with very different chemical structures, which are mostly hydrophobic and some containing hydrophilic functional group in the structure. We also interpreted that there must be a hydrogen bonding between hydrophilic OH or C=OH groups of some components in the essential oil and NH_2_ groups on the surface of PAMAM G4.0. The rest hydrophobic components tended to be entrapped within the large internal cavity of dendrimer, which is a common tendency of hydrophobic substances when being mixed with PAMAM, thus reducing the essential oil volatility. This phenomenon has been reported in many previous studies [16,26]. With the particle size of less than 500 nm, oil/PAMAM G4.0 (average diameter around 40–120 nm) is considered as a sufficiently good material for the penetration of nanocarriers into fungal cells [22,23,24,25]. In addition, PAMAM G4.0 with large number of positively charged surface –NH_2_– groups facilitates the interaction and destruction of microbial negatively charged membrane. As a result, oil/PAMAM G4.0 fully interacts with the fungus’s cell membrane as well as easily penetrates into the cell layer, thus destroying fungal cells. 

The synergistic effect in antifungal properties between the essential oils and oil/PAMAM G4.0 could be explained by the reduction of the oil volatility when it is mixed with PAMAM G4.0 structure, therefore improving the stability and maintaining the antifungal effectiveness better than essential oil in its free form. Moreover, the extremely poor water solubility of essential oil could also be improved by associating with PAMAM dendrimer, which perhaps due to the hydrogen bonds and hydrophobic interactions between the essential oils and functional groups of PAMAM G4.0. There are numerous literatures that successfully demonstrated the use of dendrimer to enhance the solubilization of hydrophobic molecules [27]. Many of such studies revealed the capability of PAMAM dendrimer in antifungal activity improvement as consequence of enhanced solubility of poorly soluble substances [28,29]. For instance, it was reported that the mixture of PAMAM G2.0 and ketoconazole, which is hydrophobic, was 16 times more potent against *Candida* in comparison with free drug [19]. 

## 4. Conclusions

In this study, TEM and ^1^H-NMR technique were employed to characterize the PAMAM G4.0 material and the marjoram essential oil was analyzed for chemical composition using GC-MS. The two samples and the oil/PAMAM G4.0 were jointly analyzed by FT-IR, showing the successful associating of oil and PAMAM G4.0 dendrimer. The resulting PAMAM G4.0@oil was shown to be more effective in terms of antifungal activity compared with PAMAM G4.0 and marjoram volatile oil with same concentration. The enhanced anti-microbial property of the oil/PAMAM G4.0 could be due to the restricted evaporation of the essential oil, caused by the encapsulation. These results suggest that the association of marjoram oil with PAMAM G4.0s is promising in combating late blight in tomatoes and that later studies should focus on determining the suitable concentration for optimal inhibition and conducting field trials.

## Figures and Tables

**Table 1:** Average diameters of inhibition zone against *Phytophthora infestants* of Marjoram essential oil, PAMAM G4.0 and oil/PAMAM G4.0 based on different concentrations: 500, 1000, 2000 and 5000 ppm.

Concentration (ppm)		Diameter of Inhibition Zone (d. mm)
Marjoram Essential Oil	PAMAM G4.0	
500	-	6.33 ± 0.33
1000	-	7.50 ± 0.33
2000	-	10.00 ± 0.33
5000	-	12.50 ± 0.50
-	500	6.16 ± 0.34
-	1000	7.50 ± 0.50
-	2000	10.00 ± 0.33
-	5000	12.00 ± 0.50
500	500	9.50 ± 0.50
1000	1000	12.33 ± 0.33
2000	2000	15.00 ± 0.00
5000	5000	18.00 ± 0.50
